# The impact of patient co-morbidities on the regenerative capacity of cardiac explant-derived stem cells

**DOI:** 10.1186/s13287-016-0321-4

**Published:** 2016-05-26

**Authors:** Audrey E. Mayfield, Megan E. Fitzpatrick, Nicholas Latham, Everad L. Tilokee, Melanie Villanueva, Seth Mount, Bu-Khanh Lam, Marc Ruel, Duncan J. Stewart, Darryl R. Davis

**Affiliations:** University of Ottawa Heart Institute, Division of Cardiology, Department of Medicine, University of Ottawa, Ottawa, K1Y4W7 Canada; Ottawa Hospital Research Institute, Division of Regenerative Medicine, Department of Medicine, University of Ottawa, Ottawa, K1H8L6 Canada

**Keywords:** Stem cell, Heart failure, Cell transplantation, Cytokine

## Abstract

**Background:**

Although patient-sourced cardiac stem cells repair damaged myocardium, the extent to which medical co-morbidities influence cardiac-derived cell products is uncertain. Therefore, we investigated the influence of atherosclerotic risk factors on the regenerative performance of human cardiac explant-derived cells (EDCs).

**Methods:**

In this study, the Long Term Stratification for survivors of acute coronary syndromes model was used to quantify the burden of cardiovascular risk factors within a group of patients with established atherosclerosis. EDCs were cultured from human atrial appendages and injected into immunodeficient mice 7 days post-left coronary ligation. Cytokine arrays and enzyme linked immunoassays were used to determine the release of cytokines by EDCs in vitro, and echocardiography was used to determine regenerative capabilities in vivo.

**Results:**

EDCs sourced from patients with more cardiovascular risk factors demonstrated a negative correlation with production of pro-healing cytokines (such as stromal cell derived factor 1α) and exosomes which had negative effects on the promotion of angiogenesis and chemotaxis. Reductions in exosomes and pro-healing cytokines with accumulating medical co-morbidities were associated with increases in production of the pro-inflammatory cytokine interleukin-6 (IL-6) by EDCs. Increased patient co-morbidities were also correlated with significant attenuation in improvements of left ventricular ejection fraction.

**Conclusions:**

The regenerative performance of the earliest precursor cell population cultured from human explant tissue declines with accumulating medical co-morbidities. This effect is associated with diminished production of pro-cardiogenic cytokines and exosomes while IL-6 is markedly increased. Predictors of cardiac events demonstrated a lower capacity to support angiogenesis and repair injured myocardium in a mouse model of myocardial infarction.

**Electronic supplementary material:**

The online version of this article (doi:10.1186/s13287-016-0321-4) contains supplementary material, which is available to authorized users.

## Background

A decade of progress towards understanding cardiac stem cell mediated repair of damaged myocardium has borne fruit with the recent completion of two phase 1 clinical trials [1, 2]. Despite this promise, significant hurdles remain before cardiac-derived cell therapy can be effectively translated to the clinic. One of the most significant barriers to this technology surrounds the effect of patient comorbidities on the regenerative capacity of ex vivo stem cells cultured directly from patient tissue specimens– the very same individuals who will likely require this therapy in the future. In other organ stem cells, increasing chronological age and co-morbidities have been shown to inhibit performance but the degree to which this translates to first generation cardiac stem cell products remains poorly defined [3].

The initial publication describing the culture of ex vivo proliferated cardiac stem cells focused primarily on cells cultured from tissue donated by post-transplant patients [4]. Although cells from the right ventricular apex of these immunosuppressed patients did not differ in crude measures of cell growth and myocardial repair/salvage, several recent publications using tissue samples sourced from non-transplant patients hint that the regenerative performance of cardiac derived cell products may be impaired by patient co-morbidities [5–8]. Unfortunately, these studies examined only crude surrogate endpoints (such as cell culture numbers and proliferation), without reference to actual myocardial repair or the potential mechanisms underlying cell-mediated cardiac repair. Recently, we demonstrated that hyperglycemia may have profound effects on the regenerative performance of cardiac stem cells which prompted us to explore the influence of other comorbidities [9].

Therefore, we investigated the influence of cardiac risk factors on the functional activity of human cardiac explant derived cells (EDCs). By focusing upon the primary outgrowth from plated cardiac samples, we elected to characterize the common early cell product used in clinical trials before antigenic sub-selection (i.e., c-Kit + cells) [10] or prolonged cell culture (i.e., cardiosphere-derived cells) [4]. Previous work has shown this early cell product provides equivalent regenerative potency while demonstrating a thousand-fold superior capacity to adopt a cardiac lineage, making EDCs the ideal cell product to investigate patient dependent effects while minimizing ex vivo culture induced artefacts [6, 9, 11–13]. Our overall hypothesis is that accumulating medical comorbidities reduce the regenerative performance of EDCs by altering the paracrine signature of these cells, which accounts for gains seen after intra-myocardial injection.

## Methods

### Patients and cell culture

Human EDCs and circulating angiogenic cells (CACs) were cultured from atrial appendages and blood samples donated by patients undergoing clinically-indicated cardiac procedures after informed consent under a protocol approved by the University of Ottawa Heart Institute Research Ethics Board [12–14]. Inclusion criteria for tissue donors selected patients between the ages of 18 and 80 who required cardiac surgery for coronary artery bypass grafting and/or valve surgery. Exclusion criteria included chronic infectious diseases (human immunodeficiency virus, hepatitis), pregnant women or active sepsis. EDCs were cultured as described previously [12, 13]. Briefly, human atrial appendages were minced, enzymatically digested and plated on fibronectin-coated dishes within cardiac explant media [Iscove’s Modified Dulbecco’s Medium (Life Technologies), 20 % fetal bovine serum (Life Technologies), 100 U/ml penicillin G, 100 ug/ml streptomycin (Life Technologies), 2 mmol/L L-glutamine (Life Technologies) and 0.1 mmol/L 2-mercaptoethanol (Life Technologies)]. EDCs were enzymatically harvested (0.05 % trypsin; Life Technologies) every 7–10 days up to three times from the same tissue sample for direct experimentation (maximal total explant culture time of 30 days). For consistency, harvests were allocated to specific experiments (i.e., Harvest 1 EDCs– survival/apoptosis experiments, harvest 2 EDCs– cell transplantation into mice and harvest 3 EDCs– generation of conditioned media). EDC conditioned media was obtained after 48 hours of culture in hypoxic (1 % oxygen) low serum (1 % serum) conditions to simulate ischemic myocardium. Cells were seeded at approximately 80 % confluency (150,000 cells per well of a six-well plate) in 2 ml of low serum media. After media collection, cells were harvested and protein lysate was obtained using radioimmunoprecipitation assay buffer (Sigma-Aldrich). Protein concentrations were measured using a Bradford assay and used to normalize results of downstream applications.

CACs were isolated from peripheral blood samples using standard culture techniques [15]. Mononuclear cells were isolated using density-gradient centrifugation (Histopaque 1077; Sigma-Aldrich) and placed in culture for 4–6 days in endothelial basal media (Clonetics, Canada) supplemented with EGM-2-MV-SingleQuots (Clonetics) that included 5 % serum, 50 ng/ml human vascular endothelial growth factor (VEGF), 50 ng/ml human insulin-like growth factor-1 (IGF-1) and 50 ng/mL human epidermal growth factor (EGF). CACs were harvested by mechanical dissociation and were used for experimentation within 7 days of culture. Commercially sourced human umbilical vein endothelial cells (HUVECs) were cultured according to the manufacturer’s directions (Lonza).

### Cytokine secretion and exosome isolation

A custom Luminex Procarta Immunoassay kit (Affymetrix, CA, USA) was used to detect cytokine concentration in the EDC conditioned media. The polystyrene conjugated beads were incubated in a 96-well plate with the conditioned media samples and the analyte standards. After a wash, the beads were incubated with a biotinylated detection antibody. After another wash the beads were incubated with Streptavidin-SE. The plate was read immediately on a Bio-Plex 200 system (Bio-Rad Technologies, CA, USA). The conditioned media samples were tested for ten analytes. Single measures of cytokine content were determined using commercial enzyme-linked immunosorbent assays (HS600B, R&D Systems). All immunosorbent measures were normalized to the protein content and media volume.

Exosomes were isolated from a separate aliquot of EDC conditioned media after 48 hours of culture in 1 % serum hypoxic conditions using ExoQuick-TC polymer-based exosome precipitation (System Biosciences). Exosome pellets were then re-suspended and exosome content was quantified using Nanoparticle Tracking Analysis (Nanosight V2.3).

### In vitro angiogenesis, cell migration and resistance to hypoxia

The ability of EDC conditioned media to stimulate angiogenesis was assessed using a growth factor depleted matrigel assay (ECM625, Millipore) according to the manufacturer’s instructions. Six random fields were analyzed and cumulative tubular growth was determined (Image J, NeuronJ plug-in; National Institutes of Health). The ability of EDC conditioned media to attract CACs was assessed using trans-well plates (24 wells, 3.0 μm pores; Corning). Serum-free DMEM containing 100 ng VEGF was used as an unbiased positive control to normalize individual variations in CAC migration, and serum-free DMEM was used as a negative control. After 24 hours of normoxic incubation, the inserts and the remaining upper compartment CACs were removed. Random field analysis was used to quantify the number of CACs (Image J, ICTN plug-in; Center for Bio-Image) that had successfully migrated through the polycarbonate membrane after fixation (4 % paraformaldehyde) and staining with 4′,6-diamidino-2-phenylindole (Sigma-Aldrich).

The ability of EDCs to resist apoptosis after 48 hours of culture in hypoxic (1 % oxygen) low serum (1 % serum) conditions was quantified using flow cytometry (Guava easyCyte 8HT, EMD Millipore) for early (annexin V) and late (7-amino-actinomycin; 7-AAD) markers of apoptosis (BD Biosciences).

All procedures and analyses were performed blinded to patient comorbidities.

### Myocardial infarction, cell injection and functional evaluation

Male non-obese diabetic severe combined immunodeficient (NOD-SCID) mice underwent surgical left coronary ligation followed 7 days later by randomization to echocardiographic guided intra-myocardial injection of 100,000 EDCs or vehicle (saline) into the infarct border and cardiac apex regions under a protocol approved by the University of Ottawa Heart Animal Care Service [9, 11, 12]. Animals were injected with buprenorphine (0.05 mg/kg; subcutaneous) 1 hour prior to surgery and twice daily thereafter for 3 days. During the ligation, mice were incubated, anesthetized using isoflurane (maintained at 2–3 %) and maintained under physiologic temperature control. Upon closure, animals were injected with 0.5 cc of saline (subcutaneous). Left ventricular ejection fraction (LVEF) was evaluated 21 and 28 days after left coronary ligation. All procedures and analyses were performed blinded to treatment allocation.

### Statistical analysis

Unless otherwise specified data are presented as mean ± standard error of the mean. Pearson’s correlation co-efficient (r) was used to assess the strength of the linear dependence between patient co-morbidities and EDC potency. A strong correlation is defined as *r* ≥ 0.5, a moderate correlation as 0.5 > *r* ≥ 0.3, a weak correlation as 0.3 > *r* ≥ 0.1, and no correlation as *r* < 0.1. Differences in categorical measures were analyzed using a χ2 test. To determine if differences existed between groups, normally distributed data were analyzed by a one-way or repeated-measures ANOVA (GraphPad Prism v.6.05); if such differences existed, Bonferroni’s corrected t-test was used to determine the group(s) with the difference(s). Non-parametric data were analyzed using Mann-Whitney or Kruskal-Wallis testing with post-hoc analysis using Dunn’s test to account for multiple comparisons. To account for multiple comparisons made from the serial echocardiograms, the functional data were analyzed using a repeated measures mixed model with post-hoc testing done using t-tests with Bonferonni’s correction. A final value of *p* ≤ 0.05 was considered significant for all analyses.

## Results

### Baseline demographics

Thirty-two patients were enrolled in this study [66 % male; age 65 ± 2 years; body mass index (BMI) 28 ± 1 kg/m^2^, Table 1]. All patients had a history of stable cardiac disease with a number of co-morbidities including hypertension (71 %), diabetes (34 %) and dyslipidemia (69 %). A number of patients had a history of coronary artery disease (75 %), previous infarct (34 %) and valvular heart disease (56 %) while very few had a history of congestive heart failure (16 %). As such, the average ventricular function was low normal (LVEF 54 ± 2 %) with only mild angina (Canadian Cardiovascular Society class 1.9 ± 0.2) and heart failure (New York Heart Association class 1.6 ± 0.1) symptoms. The majority of patients underwent elective bypass surgery alone (72 %) while the remainder underwent bypass with valve repair/replacement (15 %) or valve repair/replacement alone (13 %). All patients were on stable cardiac medications including angiotensin-converting enzyme inhibitors and/or angiotensin receptor blockers (46 %), anti-platelet therapy (78 %), beta-blockers (56 %), statins (53 %) and calcium channel blockers (43 %) for at least 3 months prior to surgery.

**Table 1:** Baseline clinical characteristics of the patients

	Number of patients (*N* = 32)	Association with advance LTS score *P* value ± *R* ^2^, as applicable
Age (yrs)	65 ± 2	0.55
BMI	28 ± 1	0.35
Gender (%male)	66 %	0.56
Hypertension	71 %	0.09
Dyslipidemia	69 %	0.12
Current smoker	9 %	0.45*
Former smoker	34 %	
Diabetes	34 %	0.0003
HbA1C	0.062 ± 0.01	0.002 (*R* ^2^ = 0.4)
Fasting glucose	6.2 ± 0.7	0.18
Thyroid or other endocrine disease	9 %	0.38
Peripheral vascular disease	25 %	0.13
History of MI	34 %	0.01
Valvular heart disease	56 %	0.09
Coronary artery disease	75 %	0.09
Congestive heart failure	16 %	0.28
NYHA class	1.6 ± 0.1	0.94
LV ejection fraction	54 ± 2	0.02 (*R* ^2^ = 0.17)
CCS class	1.9 ± 0.1	0.74
Creatinine (umol/L)	83 ± 3	0.14
Medications:		
Anti-platelet/Anti-coagulant	78 %	0.12
Beta-blocker	56 %	0.38
Statin	53 %	0.55
Diuretics	31 %	0.07
ACEI or ARB	46 %	0.11
Calcium channel blocker	43 %	0.64
Insulin	9 %	0.30
Oral hypoglycemics	31 %	<0.001

*LTS* long term stratification, *BMI* body mass index, *HbA1C* hemoglobin A1c, *MI* myocardial infarction, *NYHA* New York Heart Association, *LV* left ventricle, *CCS* Canadian Cardiovascular Society, *ACEI* angiotensin-converting enzyme inhibitors, *ARB* angiotensin receptor blockers. *p vs. LTS score in non-smokers

Given that all patients recruited into the study were undergoing clinically indicated cardiac surgery, the Long Term Stratification model for survivors of an acute coronary syndrome (LTS) was chosen as a means of discriminating between cardiac surgery patients with few or extensive comorbidities [16]. As outlined in Additional file 1: Table S1, the LTS score evaluates eight quantitative measures of health that largely reflect baseline conditions found to influence the regenerative performance of blood derived stem cells (such as advanced age, sex, diabetes and hypertension). This selection was made as the LTS risk stratification model avoids:1) cumbersome lab testing [17, 18], 2) unstable patient subsets [19], 3) geographical or social biases [20, 21], and 4) clustering of an inherently biased cardiac surgery population within a high risk category alone [22]. We observed that the distribution of LTS scores within the population of patients who donated atrial appendages ranged from −3 to 12 ($\overline{x}$ = 3.8, standard deviation = 3.3; Additional file 1: Figure S1). As expected, increasing LTS score was associated with several well-known markers for increased risk of cardiac events including advanced hemoglobin A1c score (*R*^2^ = 0.4, *r* = 0.002 ± 0.0004, *p* = 0.0006), reduced left ventricular ejection fraction (*R*^2^ = 0.17, *r* = −1.5 ± 0.6, *p* = 0.02), a history of diabetes, and a history of myocardial infarction.

### EDC regenerative performance inversely correlates with risk factors for future cardiac events

**Fig. 1:**
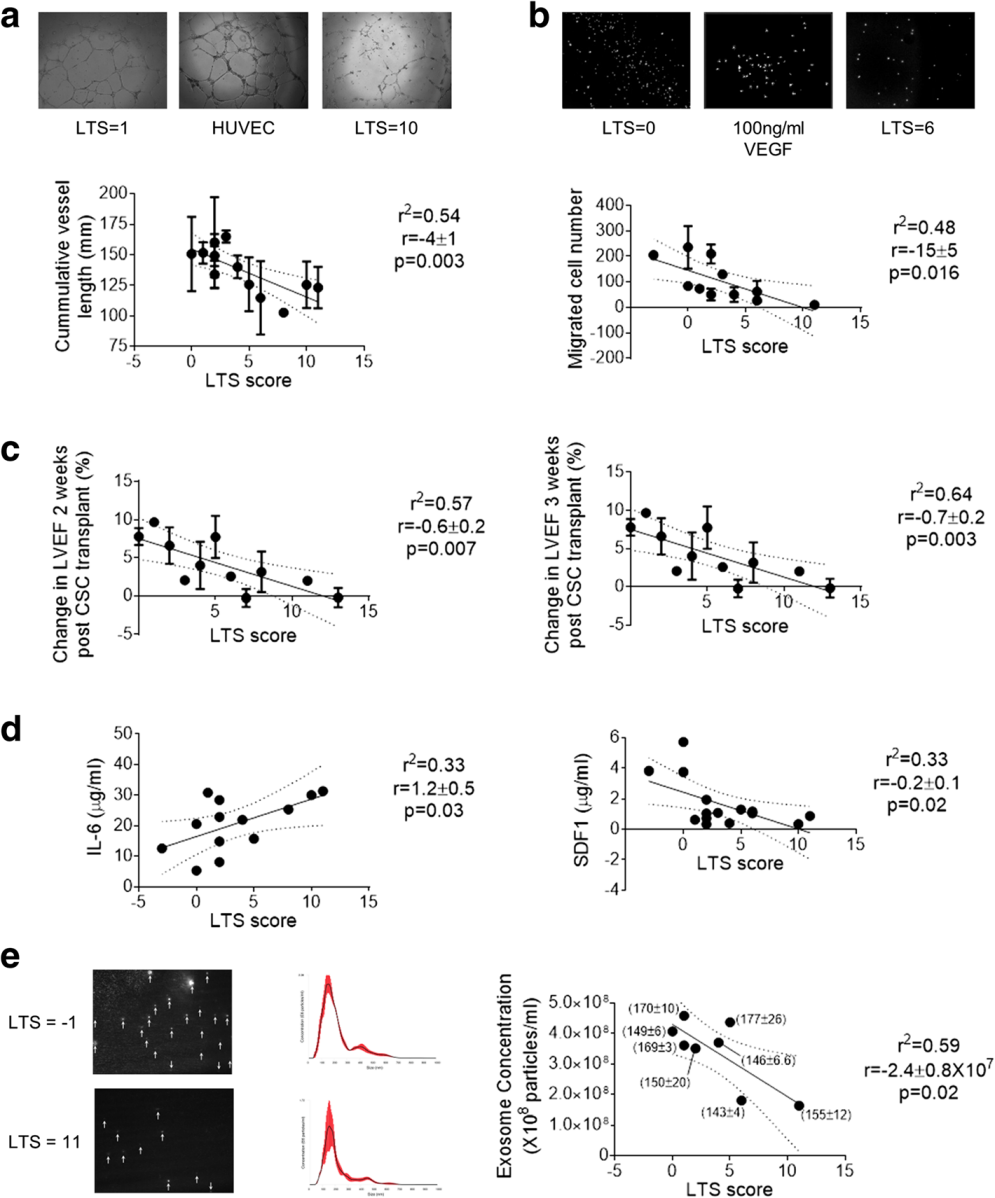
Relationship between the long-term risk of coronary heart disease events and the regenerative performance of EDCs. **a** Representative images of HUVEC tubules formed after exposure to media conditioned by EDCs sourced from patients with variable LTS scores. Correlation between patient LTS score and the ability of EDC conditioned media to stimulate HUVEC tubule formation (*n* = 13). **b** Representative images of 4′,6-diamidino-2-phenylindole-stained circulating angiogenic ells (CACs) after 24 hours exposure to media conditioned by EDCs sourced from patients with variable LTS scores. Correlation between patient LTS score and the ability of EDC conditioned media to recruit CACs across a transwell membrane (*n* = 11). **c** Correlation between patient LTS score improvements in echocardiographic left ventricular ejection fraction (LVEF) measured 2 and 3 weeks after intramyocardial injection into an immunodeficient mouse model of myocardial ischemia (*n* = 37). **d** Correlation between LTS score and cytokine content within EDC conditioned media (IL-6, interleukin 6, *n* = 13; SDF-1α, stromal-derived factor 1 alpha; *n* = 15). **e** Representative screen capture images of media conditioned by EDCs from patients with high and low LTS score (*white arrows* indicate exosomes). Correlation between LTS score and number of exosomes within EDC conditioned media (*n* = 8). Also shown adjacent to each point is the mode ± SEM size distribution (*n* = 3 technical repeats per sample). *EDCs* explant derived cells, *HUVEC* human umbilical vein endothelial cell, *LTS* long term stratification

Accumulating medical co-morbidities demonstrated a strong inverse correlation with the ability of EDC conditioned media to stimulate angiogenesis (*R*^2^ = 0.53; r = −4 ± 1, *p* = 0.003; Fig. 1a) and recruit CACs across a transwell filter (*R*^2^ = 0.48; *r* = −15 ± 5, *p* = 0.016; Fig. 1b). These in vitro effects where mirrored in an immunodeficient mouse model of myocardial ischemia as the LVEF demonstrated a strong inverse correlation with increasing cardiovascular risk factors at both 2 (*R*^2^ = 0.57, *r* = −0.6 ± 0.2, *p* = 0.007) and 3 (*R*^2^ = 0.64, *r* = −0.7 ± 0.2, *p* = 0.003) weeks after EDC transplant (Fig. 1c). Similarly, treatment of mice with EDCs from patients with increasing cardiovascular risk factors demonstrated a strong correlation with scar burden (*R*^2^ = 0.59, *r* = −0.5 ± 0.2, *p* = 0.04, data not shown).

Of the ten cytokines analyzed using the multiplex assay, eight cytokines were detected in all of the samples tested (Fig. 1d and Additional file 1: Figure S2). The most abundant cytokine was the pro-inflammatory cytokine IL-6 with levels 10 fold greater than vascular endothelial growth factor (VEGF; 20.2 ± 2.9 vs. 2.0 ± 0.7 μg/ml, *p* < 0.05), the next most abundant cytokine. When plotted against the risk of future events as indicated by the LTS score, a moderate negative correlation was observed for stromal cell derived factor 1 alpha (SDF-1α; *R*^2^ = 0.33; *r* = −0.2 ± 0.1, p = 0.02) while a moderate positive correlation was seen with IL-6 (*R*^2^ = 0.33, *r* = 1.2 ± 0.5, *p* = 0.03) levels. There was no significant correlation between LTS score and the remaining six cytokines (Additional file 1: Figure S2). These findings highlight cytokine variations previously observed in media conditioned by EDCs sourced from patients with diabetes (i.e., IL-6) [9] and heart failure (i.e., SDF-1α) [23].

Given recent evidence that a portion of cardiac repair after EDC transplant may in part be attributable to the production of exosomes [24], the number of exosomes within media following low serum hypoxic culture was quantified. This analysis demonstrated a moderate correlation between LTS score and the number of exosomes within conditioned media (Fig. 1e; *R*^2^ = 0.59; *r* = −2.4 ± 0.8 × 10^7^, *p* = 0.02); suggesting that EDCs sourced from patients with accumulating medical co-morbidities produced progressively fewer exosomes.

Flow cytometry of EDCs after 2 days of culture in hypoxic low serum conditions demonstrated that progressive LTS score did not predict changes in early markers of apoptosis (annexin V; *R*^2^ = 0.03, *r* = 1.9 ± 5.9, *p* = 0.73), markers of late apoptosis (annexin V + 7AAD, *R*^2^ = 0.04, *r* = 0.1 ± 0.3, *p* = 0.68), or markers of necrosis (7AAD, *R*^2^ = 0.04, *r* = 1.3 ± 0.3, *p* = 0.70).

These results suggest that EDCs from patients with more cardiovascular risk factors have a lower capacity to support angiogenesis and repair injured myocardium. Given recent evidence that exosomes and SDF-1α promote indirect cardiac repair by transplanted EDCs, the findings seen in this study may in part be ascribed to the observed decline in exosome and SDF-1α production [23]. Although the changes observed in exosomes and SDF-1α were notable, the modest disparity in exosome (Δ-1.9 × 10^8^ particles/ml) and SDF-1α (Δ-3.5 μg/ml) content was dwarfed by differences seen in IL-6 content (Δ + 17 μg/ml) hinting that this pro-inflammatory cytokine may be playing a key role in indirect cardiac repair by EDCs.

Although diabetes is clearly associated with impaired EDC-mediated repair of myocardium [9] and greater LTS score, poorer glycemic control (i.e., greater HbA1c) alone did not linearly correlate with changes in IL-6 (*p* = 0.69), SDF1α (*p* = 0.43) and exosome production (*p* = 0.82). Taken together, these data highlight the additional value in considering the many factors that predict future cardiac events and autologous stem cell dysfunction.

## Discussion

Although cardiac stem cells have swiftly advanced to clinical trials, the degree to which patient comorbidities limit regenerative capacity has yet to be fully investigated. In this study, we found that, despite using a biased patient population (i.e., patients undergoing clinically-indicated cardiac surgery), an increased burden of cardiovascular co-morbidities reduces the ability of EDCs to promote post-infarct repair. This reduction may be mediated by diminished production of pro-healing exosomes [24] and cytokines [23, 25] but was also intriguingly associated with enhanced expression of the pro-inflammatory cytokine IL-6.

Unlike previous studies examining the influence of clinical characteristics on non-cardiac stem cells [3], autologous cardiac-derived cell therapy necessitates invasive cardiac biopsy which effectively precludes harvest from normal individuals not undergoing invasive cardiac procedures. After a survey of 18 risk scoring systems, we identified the LTS score as a simple means of discriminating between patients with established cardiovascular risk factors. Advanced LTS score was shown to predict diminished responses within established in vitro [26–28] and in vivo [4, 10, 29] assays of stem cell performance.

Analysis of the broad array of cytokines known to be expressed by EDCs [12, 13] demonstrated that only SDF-1α declined with progressive LTS score– a finding that highlights evidence previously showing that SDF-1α may play an important role in promoting post infarct healing by EDCs [23, 30, 31]. However, diminished production of cytokines alone cannot account for the effects observed in EDCs sourced from patients with advanced LTS scores as evidenced by the number of cytokines that remained unaffected by medical comorbidities. This prompted investigations that demonstrated the overall production of exosomes was reduced by EDCs with increasing LTS scores- an observation in keeping with accumulating evidence that exosomes contribute to post infarct myocardial repair [24].

Patients with diabetes and poor glycemic control (i.e., worse HbA1c) inherently had greater LTS scores as diabetes is a key predictor of future cardiac events. However, as shown above, poor glycemic control alone was not sufficient to predict reduced EDC function. The inclusion of multiple other medical issues enhanced our ability to predict EDC dysfunction and was associated with clear changes in the in vitro regenerative performance of EDCs. To enable customized solutions for autologous therapy in patients with multiple medical illnesses, future work needs to be directed towards dissecting the fundamental mechanisms underlying the observed effect.

## Conclusions

This study provides a greater understanding of the relationship between clinical markers of disease burden and the regenerative performance of EDCs, a result likely attributable in part to reduced exosome and cytokine production. Enhanced production of IL-6 by patients with extensive co-morbidities hints that this key pro-inflammatory cytokine may be playing a role in EDC-mediated repair- although this remains to be shown. By defining the variables that underlie the regenerative potential of EDCs, this research opens new avenues towards understanding and improving stem cell therapeutics.

## Additional file

Additional file 1: Figure S1. Histogram of LTS frequency distribution in patient samples used to culture EDCs for experimentation. **Figure S2.** Effect of patient LTS score on cytokine production. Correlation between LTS score and cytokine content within EDC conditioned media (FGF, fibroblast growth factor, n = 16; HGF, heptocyte growth factor, n = 16; PDGF, platelet derived growth factor, n = 15 EDC cell lines; SCF, stem cell factor, n = 16 EDC cell lines; TNF, tumor necrosis factor, n = 16 EDC cell lines; VEGF, vascular endothelial growth factor, n = 13 EDC cell lines). **Table S1.** Comparison of outcomes assessed in different quantitative measures of health scoring systems. (DOCX 339 kb)

## Abbreviations

7-AAD: 7-amino-actinomycin
ACEI: angiotensin-converting enzyme inhibitors
ARB: angiotensin receptor blockers
BMI: body mass index
CACs: circulating angiogenic cells
CCS: Canadian Cardiovascular Society
EDCs: explant-derived cells
EGF: epidermal growth factor
HbA1C: hemoglobin A1c
HUVECs: human umbilical vein endothelial cells
IGF-1: insulin-like growth factor-1
IL-6: interleukin-6
LTS: long term stratification model for survivors of an acute coronary syndrome
LV: left ventricle
LVEF: left ventricular ejection fraction
MI: myocardial infarction
NOD-SCID: non-obese diabetic severe combined immunodeficient
NYHA: New York Heart Association
VEGF: vascular endothelial growth factor
