# Supplementary material for: The impact of patient co-morbidities on the regenerative capacity of cardiac explant-derived stem cells
Source: Stem Cell Res Ther. 2016 May 26;7:60. doi: 10.1186/s13287-016-0321-4 (PMC4880978; doi:10.1186/s13287-016-0321-4)
Supplement: Additional file 1: Figure S1. — Histogram of LTS frequency distribution in patient samples used to culture EDCs for experimentation. Figure S2. Effect of patient LTS score on cytokine production. Correlation between LTS score and cytokine content within EDC conditioned media (FGF, fibroblast growth factor, n = 16; HGF, heptocyte growth factor, n = 16; PDGF, platelet derived growth factor, n = 15 EDC cell lines; SCF, stem cell factor, n = 16 EDC cell lines; TNF, tumor necrosis factor, n = 16 EDC cell lines; VEGF, vascular endothelial growth factor, n = 13 EDC cell lines). Table S1. Comparison of outcomes assessed in different quantitative measures of health scoring systems. (DOCX 339 kb) [file 13287_2016_321_MOESM1_ESM.docx]

**Additional file 1**

**Figure S1. Histogram of LTS frequency distribution in patient samples used to culture EDCs for experimentation.**


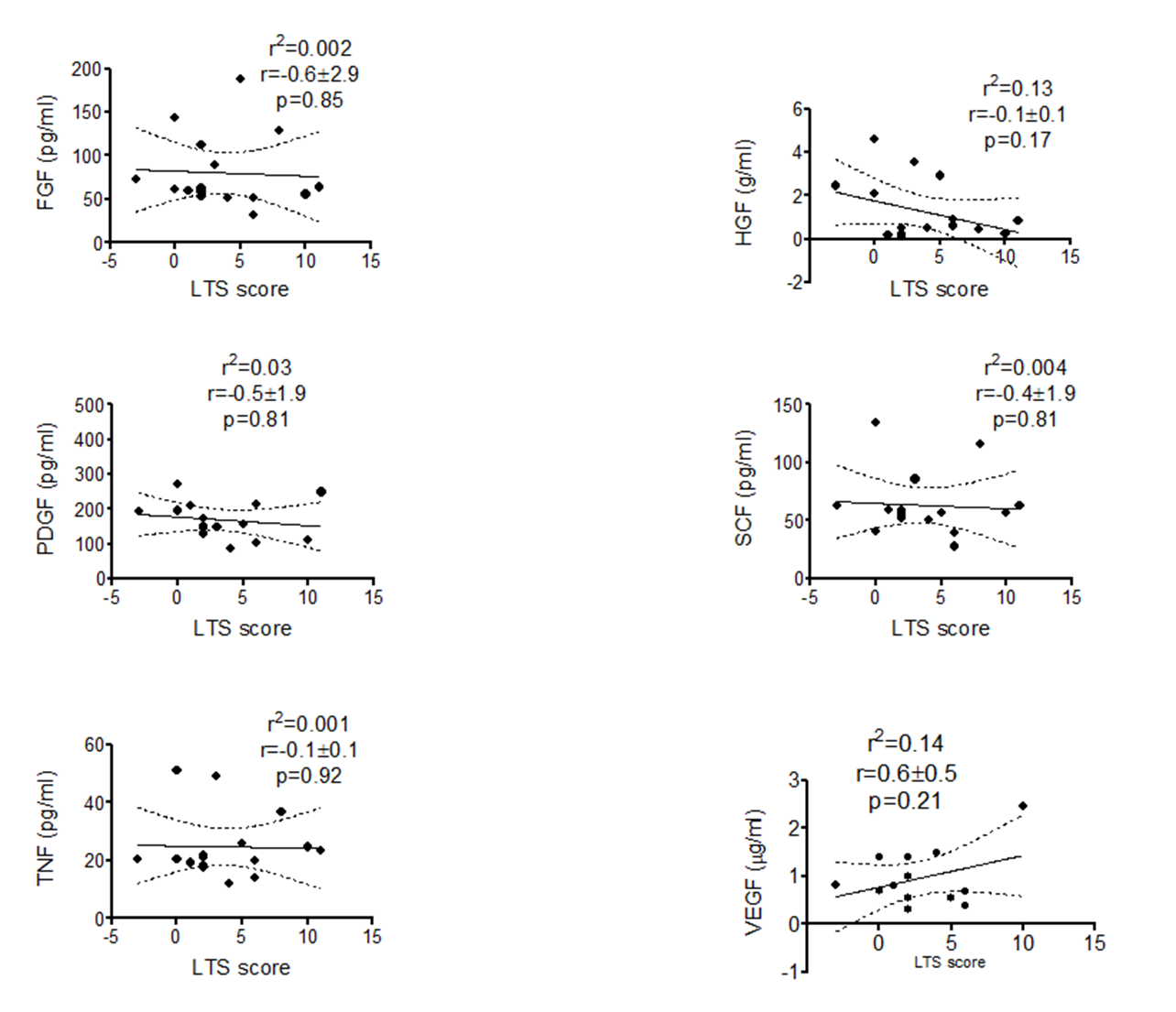


**Figure S2. Effect of patient LTS score on cytokine production.** Correlation between LTS score and cytokine content within EDC conditioned media (FGF, fibroblast growth factor, n=16; HGF, heptocyte growth factor, n=16; PDGF, platelet derived growth factor, n=15 EDC cell lines; SCF, stem cell factor, n=16 EDC cell lines; TNF, tumor necrosis factor, n=16 EDC cell lines; VEGF, vascular endothelial growth factor, n=13 EDC cell lines).


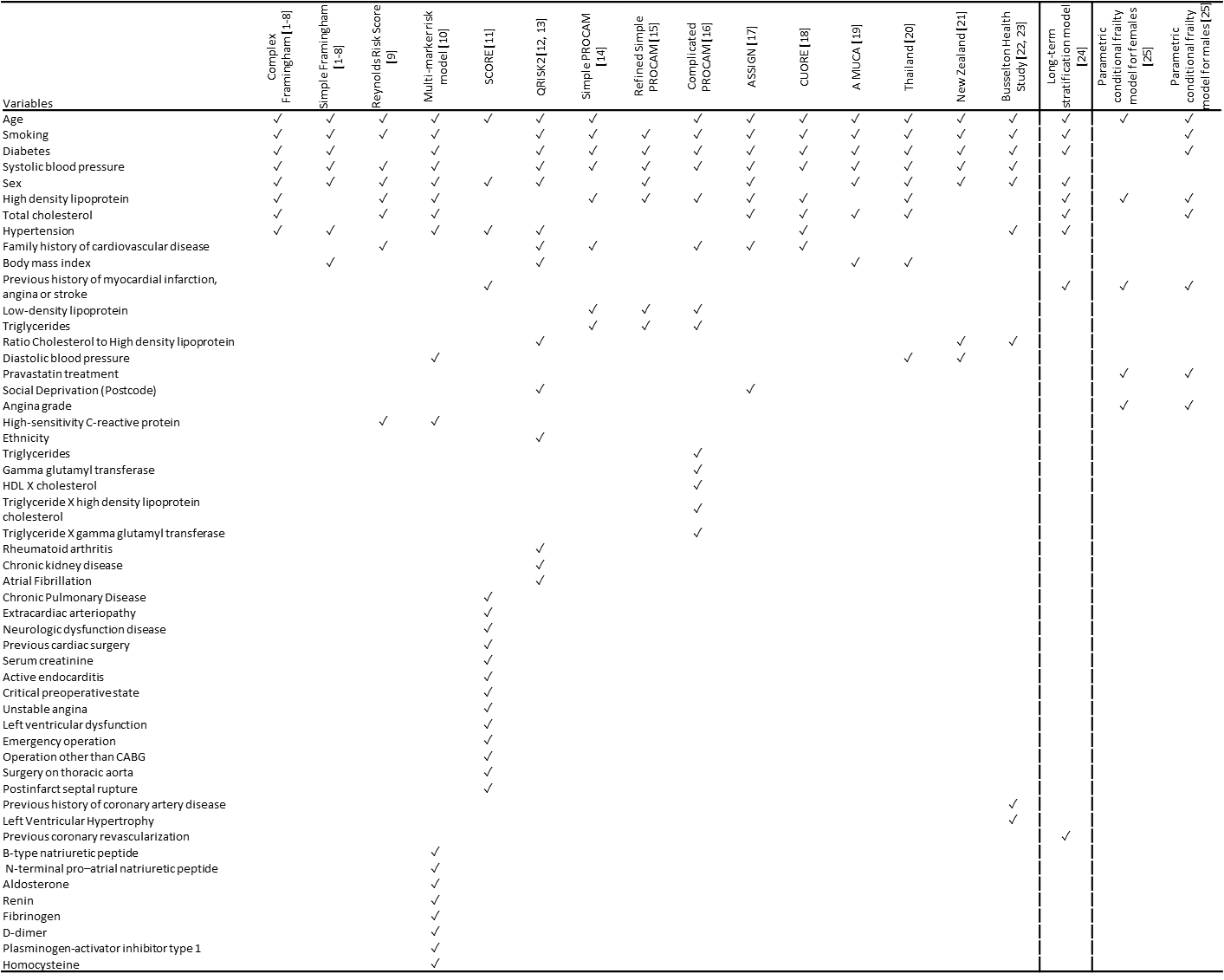


**Table S1. Comparison of outcomes assessed in different quantitative measures of health scoring systems.**
